# Construction of a Full-Length Enriched cDNA Library and Preliminary Analysis of Expressed Sequence Tags from Bengal Tiger *Panthera tigris tigris*

**DOI:** 10.3390/ijms140611072

**Published:** 2013-05-24

**Authors:** Changqing Liu, Dan Liu, Yu Guo, Taofeng Lu, Xiangchen Li, Minghai Zhang, Jianzhang Ma, Yuehui Ma, Weijun Guan

**Affiliations:** 1 Institute of Animal Science, Chinese Academy of Agricultural Sciences, Beijing 100193, China; E-Mails: lcq7813@hotmail.com (C.L.); taofenglu@yahoo.com.cn (T.L.); nobelli@126.com (X.L.); Yuehui.ma@263.net (Y.M.); 2 Department of Bioscience, Bengbu Medical College, Bengbu 233000, China; E-Mail: ily0720@126.com; 3 The Northeast Tiger Wooden Land of Heilongjiang, Harbin 150028, China; E-Mail: liudan_1964@sina.com; 4 College of Wildlife Resource, Northeast Forestry University, Harbin 150028, China; E-Mails: zhangminghai2004@126.com (M.Z.); jianzhangma@163.com (J.M.)

**Keywords:** Bengal tiger, *Panthera tigris tigris*, fibroblast cell line, SMART cDNA library, expressed sequence tags

## Abstract

In this study, a full-length enriched cDNA library was successfully constructed from Bengal tiger, *Panthera tigris tigris*, the most well-known wild Animal. Total RNA was extracted from cultured Bengal tiger fibroblasts *in vitro*. The titers of primary and amplified libraries were 1.28 × 10^6^ pfu/mL and 1.56 × 10^9^ pfu/mL respectively. The percentage of recombinants from unamplified library was 90.2% and average length of exogenous inserts was 0.98 kb. A total of 212 individual ESTs with sizes ranging from 356 to 1108 bps were then analyzed. The BLASTX score revealed that 48.1% of the sequences were classified as a strong match, 45.3% as nominal and 6.6% as a weak match. Among the ESTs with known putative function, 26.4% ESTs were found to be related to all kinds of metabolisms, 19.3% ESTs to information storage and processing, 11.3% ESTs to posttranslational modification, protein turnover, chaperones, 11.3% ESTs to transport, 9.9% ESTs to signal transducer/cell communication, 9.0% ESTs to structure protein, 3.8% ESTs to cell cycle, and only 6.6% ESTs classified as novel genes. By EST sequencing, a full-length gene coding ferritin was identified and characterized. The recombinant plasmid pET32a-TAT-Ferritin was constructed, coded for the TAT-Ferritin fusion protein with two 6× His-tags in *N* and *C*-terminal. After BCA assay, the concentration of soluble Trx-TAT-Ferritin recombinant protein was 2.32 ± 0.12 mg/mL. These results demonstrated that the reliability and representativeness of the cDNA library attained to the requirements of a standard cDNA library. This library provided a useful platform for the functional genome and transcriptome research of Bengal tigers.

## 1. Introduction

Tiger (*Panthera tigris Linnaeus*, 1758) is a special species only found in Asia and considered as a symbol of beauty, power, and bravery [1]. There are four generally accepted tiger subspecies in China, Siberian tigers (*P. t. altaica*), Indochinese tigers (*P. t. corbetti*), South China tigers (*P. t. amoyensis*), and Indian or Bengal tigers (*P. t. tigris*). Today an estimated fewer than 4580 Bengal tigers survive in Bangladesh, Nepal, eastern India, Bhutan, Burma and southwest China, and less than 30 now exist in China [2,3].

The tiger is warranted the highest level of protection by the Convention on International Trade in Endangered Species of Wild Fauna & Flora (CITES). In 1989, the Chinese government placed the Bengal tiger into category I of National Protected Animal breed. In order to preserve the wealth of biodiversity in China, and to uncover the complex underlying interactions between organisms and environment, there is a very urgent need to commence rigorous conservation of endangered species. The study of full-length cDNAs remains an indispensable approach for structural and functional genome annotations. However, up to now, only about 25 functional genes and EST sequences of *Panthera tigris* have been cloned and partially studied according to the latest data of NCBI, and information with reference to cDNA library of *P. t. tigris* is scarce. To identify more genes of Bengal tiger, including the characterization of specific expressed, new or unknown functional genes and further study of their functions, construction of full-length cDNA libraries of Bengal tiger is an efficient method [4,5].

In this paper, we have cryopreserved Bengal tiger this international protected genomic resource at cellular level by establishing Bengal tiger fibroblasts cell line for the purpose of providing a convenient and effective resource for genome and transcriptome research. Meanwhile, a study on a normalized full-length cDNA library construction and preliminary analysis of ESTs from Bengal tiger fibroblast cells conducted in our laboratory is hereby described.

## 2. Results

### 2.1. Cell cultures and Characteristic Tests

**Figure 1:**
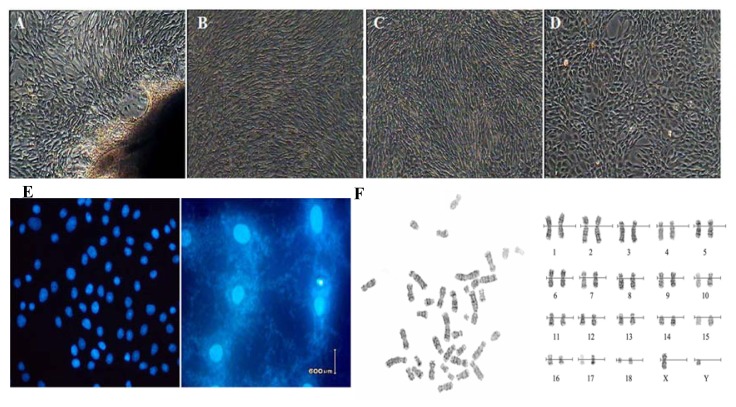
Morphology, Mycoplasma contamination and karyotype of Bengal tiger cell line. (**A**) Primary cells (×100), the cells were typical long spindle-shape; (**B**) Subcultured cells (×100); (**C**) Cells before cryopreservation (×100); (**D**) Cells after recovery (×100); (**E**) Mycoplasma contamination stained with Hoechst33258 and positive control of Mycoplasma contamination; (**F**) G-band chromosome at metaphase (**Left**) and karyotype (**Right**) (♂, ×1,000).

We used a primary explanting technique and cell cryogenic preservation technology to establish the Bengal tiger fibroblast cell line and proceeded to biological and genetic detection. The culture conditions were optimal, and the cells were healthy (Figure 1A–D). The test results of the bacteria, virus and Mycoplasma were negative (Figure 1E). To conserve genomic character of Bengal tiger, the fibroblast must maintain diploid character similar with the cells *in vivo*. Chromosome analysis showed that the frequency of cell chromosome number of 2*n* = 38 was 90.6%–92.2% in passages 1 to 3, which indicated that culture *in vitro* effects the heritage of cells slightly, supporting the theory that the cell line was a steady diploid one(Figure 1F).

### 2.2. Total RNA Extraction and LD-PCR

**Figure 2:**
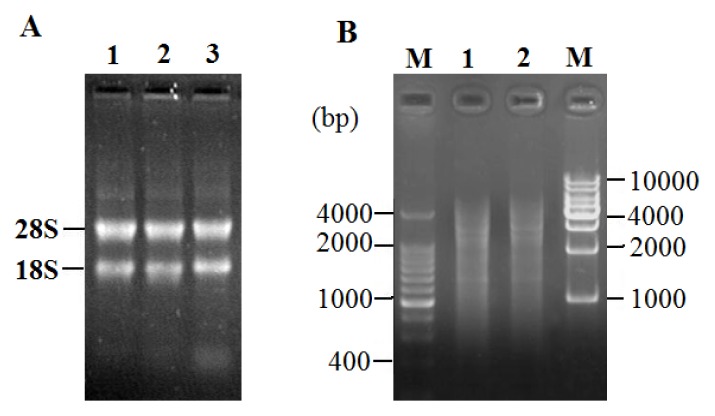
Total RNA from fibroblast cells of Bengal tiger and long distance PCR (LD-PCR). (**A**) Total RNA from fibroblast cells of Bengal tiger. Lane 1: a sample of 5 μL total RNA; Lane 2, 3: two samples incubated at 37 °C for 2 h and 3 h, respectively; (**B**) The products of LD-PCR. M: marker; Lane 1, 2: the products of LD-PCR with 22 cycles.

The ratio of OD_260_/OD_280_ for total RNAs was approximately 1.96–2.08 and the concentrations were 0.926–1.231 μg/μL. As shown in Figure 2A, two bright bands of 18*S* rRNA and 28*S* rRNA can be seen clearly, indicating that the total RNA was pure, integrated and stable enough for cDNA library construction. The stability of RNA was verified by incubating a small sample at 37 °C for two and three hours. There were little differences among the incubated and the fresh samples. Two micrograms of total RNA was subjected to reverse transcription for synthesis of the first and double-stranded cDNAs for LD-PCR. As shown in Figure 2B, the ds-DNA appeared as a smear of bands of 0.5–4 kb on the gel.

### 2.3. Characterization of cDNA Library

**Figure 3:**
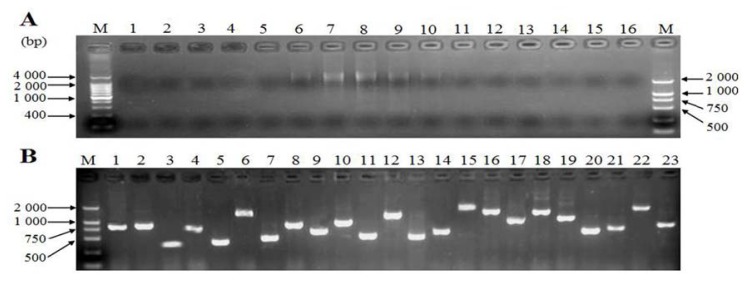
cDNA size fractionation and Recombinant clones screening. (**A**) cDNA size fractionation by CHROMA SPIN-400. M: DNA marker; 1–16: tube serial number; (**B**) Recombinant clones screening within the library. M: DNA marker; 1–23: PCR products for clones selected randomly.

cDNA-fragments smaller than 500 bp and longer than 4000 bp were eliminated by cDNA fractionation using a CHROMA SPIN-400 column to avoid the library having a preponderance of very small inserts and/or non-recombinant clones(Figure 3A). The titers of primary and amplified libraries were 1.28 × 10^6^ pfu/mL and 1.56 × 10^9^ pfu/mL respectively. The recombination efficiency of the amplified libraries was 90.2%. The insert ratio and the average length of inserted fragments were measured by PCR, as shown in Figure 3B. The average size was approximately 0.98 kb, 1–2 kb in 58.8% and 0.5–1.0 kb in 38.3%, suggesting that the insertion fragments harbored most of the mRNAs and reached the requirement for further studies on gene structure, translation, and expression.

### 2.4. Generation of Expressed Sequence Tags and Sequence Analysis

The primary cDNA library, instead of the amplified library, was used for generation of ESTs to reduce the redundancy of cDNA clones as only a small number of ESTs were targeted through random selection. Four hundred and fifty-six white clones were picked randomly for EST sequencing. After removal of the vector sequences and low-quality sequences, 354 effective sequences from the total cDNA sequences were obtained, a total of 212 individual ESTs ranging from 356 to 1108 nucleotides in length were analyzed and partly deposited in the GenBank under accession No. from JZ331652 to JZ331708.

**Figure 4:**
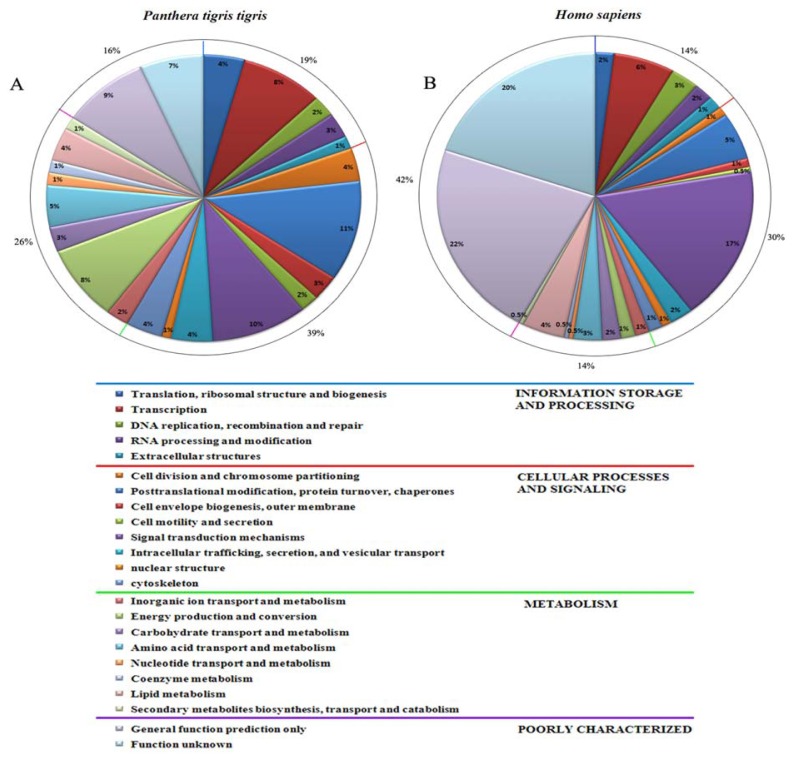
Comparison of classification of ESTs from *P. t. tigris* cDNA library based on their putative functions with those of *H. sapiens.* Two cDNA data sets were classified into functional groups by using the Clusters of Orthologous Group (COG) database.

The distribution of ESTs from *P. t. tigris* cDNA library revealed that 108 (48.1%) of them were classified as strong matches to sequences in the non-redundant protein database (Nr) for the highest match with a *E*-value less than 10^−25^, meanwhile 96 (45.3%) ESTs were nominal with *E*-value for the highest match between 10^−10^ and 10^−25^, and 14 (6.6%) ESTs were weak with *E*-value for the highest match greater than 10^−10^ or no significant similarity to sequences in the database. Based on identification of Clusters of Orthologous Group of protein (COGs), 212 ESTs were assigned to COGs by BLASTX. The proportion pattern of each COG subcategory was similar between *P. t. tigris* and *Homo sapiens.* Among the ESTs with known putative function, 56 (26.4%) ESTs were found to be related to all kind of metabolisms, 41 (19.3%) ESTs to information storage and processing, 24 (11.3%) ESTs to posttranslational modification, protein turnover, chaperones, 24 (11.3%) ESTs to transport, 21 (9.9%) ESTs to signal transducer/cell communication, 19 (9.0%) ESTs to structure protein, 8 (3.8%) ESTs to cell cycle, and only 14(6.6%) ESTs were “unknown protein” with no significant matches or unknown proteins (Figure 4).

### 2.5. Cloning and Sequence Analysis of Bengal Tigers Ferritin cDNA

**Figure 5:**
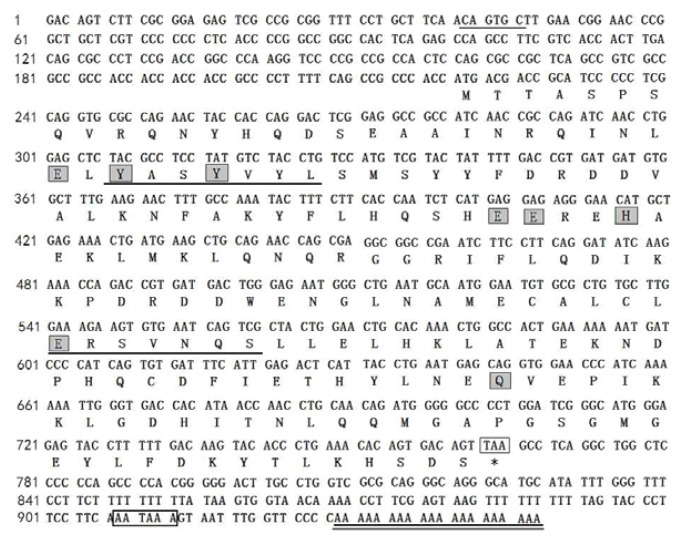
Nucleotide and deduced amino acid sequences of *Panthera tigri*s ferritin subunit (GenBank accession No.GQ891094). Seven boxed residues represented a tentatively active site of ferroxidase. The polyadenylation signal was in bold and the poly (A) tail was underlined. Iron associated residue (Try30) was in shaded square.

The full-length ferritin cDNA was 948 bp, including a 5′-untranslated region (UTR) of 219 bp, a 3′-noncoding region of 183 bp with a canonical polyadenylation signal sequence AATAAA and a poly (A) tail, and an open reading frame (ORF) of 546 bp encoding a polypeptide of 181 amino acids (Figure 5). The molecular weight of the protein was 21116.6 Mr, and the theoretical isoelectric point 5.66. The 5′-UTR contained a putative iron responsive element (IRE) sequence (−189 to −157, TCCTGCTTCAACAGTGCTTGAACGGAACCCGGC), which could be folded into a stem-loop structure for the binding site of iron regulatory protein (IRP), indicated that the expression of *P.tigris* ferritin was regulated at the translational level by iron. One stem loop structure was found in the 5′-UTR, which consisted of a six-nucleotide loop (CAGUGC) in agreement with the structures from other species such as *Homo sapiens* H [6], *Bos Taurus* [7], *Equus caballus* [8] and *Sus scrofa* [9]. The ferritin of the *Panthera tigris* has two conserved metal binding sites of the ferritin family members YASYV (T) YL (M,Q) and EK (R) S (N) VNQS.

### 2.6. Expression and Purification of Trx-TAT-Ferritin Fusion Protein

**Figure 6:**
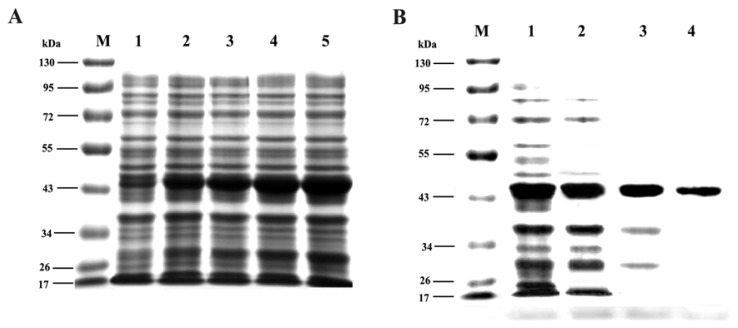
The expression and purification of Trx-TAT-Ferritin recombinant protein. (**A**) Protein expression of Trx-TAT-Ferritin with different induction time on 1, 2, 4, 6 and 8 h; (**B**) Trx-TAT-Ferritin purification under native conditions, 1. Flow through, 2–4. first to third column volume.

The final plasmid of pET32a-TAT-Ferritin, coded for the TAT-Ferritin fusion protein with two 6× His-tags in N and C-terminal, was constructed. The expression of Trx-TAT-Ferritin recombinant protein increased with the extension of culture time (Figure 6A). Samples obtained from each purification step were subjected to SDS-PAGE. Approximately 45 kDa of protein band corresponded in size to the full-length form of Trx-TAT-Ferritin fusion protein. The optimal induction requirement for the recombinant protein was 0.1 mM for 6 h. After induction for 6 h, the expression media of Trx-TAT-Ferritin was concentrated to 50 fold by resuspending the pellet in 4 mL of BugBuster reagent, the tagged proteins were purified through Ni–NTA His•Bind Resin under native conditions, the larger number of soluble proteins appeared in the first wash, and declined gradually in the following steps (Figure 6B). After BCA assay, the concentration of Trx-TAT-Ferritin recombinant protein was 2.32 ± 0.12 mg/mL.

## 3. Discussions

Over the past years, cDNA library construction and analysis is considered to be an indispensable tool for functional genome analysis as it provides much more detailed information on the genomic mechanisms underlying diverse processes of the organism [10]. The major characteristic of cDNA construction by SMART technique could improve the ratio of full-length cDNA sequences. Although the optimal number of cycles for minimizing PCR-induced mutations has not been rigorously tested for SMART library construction, performing three cycles using a suitable polymerase mixture should be sufficient to limit the number of errors to a tolerable level [11,12]. An important characteristic of SMART technique is that it provides a method for producing high-quality and full-length cDNA libraries that preserve the complete 5′ terminal sequence of mRNA [13].

### 3.1. Characterization of cDNA Library of Bengal Tiger

There are three chief aspects that identify the quality of a cDNA library. According to Clareke-Carbon’s formula, a cDNA library should contain at least 1.7 × 10^5^ independent clones to ensure that the 99% low abundance mRNA would be present in the library [14]. The high recombination efficiency is another index of good quality library [15,16]. The third aspect is that the average length of inserted cDNA should be no less than 1.0 kb to ensure the integrity of cDNA. Because selection bias could favor the smaller cDNA, we used fewer PCR cycles to minimize such bias as previously suggested [17]. In our study, up to 22 PCR amplification cycles were used to generate adequate amounts of cDNA for cloning. Most of the cDNA inserts ranged from 500 to 1500 bp, and there were a high number of cDNA clones harboring inserts over 3000 bp. Such results indicated that the size fraction was an effective selection approach to ensure the full-length cDNA content level in the cDNA library.

The diploid fibroblast cells may be injured and changed in biological characteristics, especially hereditary characteristics, after too many passages and trypsin digestion. Improving culture procedure and decreased the passages (<4) to maintain fibroblast sample character similar with the cells *in vivo*, we constructed the cDNA library to conserve genomic characteristics of Bengal tiger. The cDNA library constructed in this study will be affluent in the EST library, full-length analysis, the further work of expression identification, location and function in the chromosome will make great promotion for interesting genes associated with its excellent characters of Bengal tiger.

### 3.2. Generation and Analysis of ESTs

cDNA libraries are widely used to identify genes and splice variants, and as a physical resource for full-length clones[18,19]. The SMART library provided a useful resource for the functional genomic research of Bengal tiger and would present some new molecular material for this species as well. Generation of ESTs is an excellent and unique approach in molecular studies as it allows both expression and measurements, and the discovery of new genes to be conducted at the same time. Consequently, analysis of the expression of a large number of genes combined with the knowledge of their functions can facilitate the understanding and allows us to take a glimpse of the overall picture of biological processes in *P. t. tigris* fibroblast cells. In this study, approximately 93.4% of the ESTs generated were sequences with known or putative functions, while the remainder was unknown proteins or sequences with no similarities to the databases. These unknown and unclassified ESTs can become candidates for discovering new interesting genes through functional analysis currently being initiated in our laboratory.

### 3.3. Expression and Purification of Trx-TAT-Ferritin Fusion Protein

Recently, TAT technology has become a powerful tool for basic research, and several studies have demonstrated its usefulness for studying the role of proteins, or for targeting specific protein/protein interactions *in vitro* as well as *in vivo*. The results in our study demonstrated that Trx-TAT-Ferritin protein was highly expressed after induction for 6 h, with relative molecular weight of 45 kDa. In addition, the expressed product was present in a native and soluble form and the yield of the purified fusion protein were about 2.32 ± 0.12 mg/mL. The purified fusion protein provided valuable materials to induce the ferritin protein truncation and expression in cell.

## 4. Materials and Methods

### 4.1. Material and Reagents

Bengal tiger ear tissue samples (12 male and 10 female) were sampled from The Northeast Tiger Wooden Land of Heilongjiang.

### 4.2. Cell Cultures and Biological Analysis

Bengal tiger ear tissue samples (about 1 cm^2^ in size) were chopped finely into 1 mm^3^ in size and added DMEM medium with 10% fetal bovine serum in a 37 °C incubator with 5% CO_2_ to culture fibroblast line. Characteristic tests for established cell line with cell viability, microorganism detection and chromosome analysis: for details of the procedure used see Liu *et al.* [20].

### 4.3. cDNA Library Construction

Cells were harvested and total RNA was extracted with Trizol reagent (Invitrogen, Carlsbad, CA, USA) when they were in the period of passage 3. First and double-strand cDNAs were synthesized according to the protocol of the SMART cDNA Library Construction kit (Clontech, Palo Alto, CA, USA). Subsequently approximately 2 μL of first strand cDNA sample was amplified using long distance PCR (LD-PCR). The first four peak fractions containing cDNA (>500 bp) were pooled together using column chromatograph with CHROMA SPIN-400 medium. The cDNA was ligated to λTriplEx2 vector (1:1.5) and the ligation was packaged with Gigapack III Gold Packaging extract.

### 4.4. Titration of the Primary Library

The number of clones was counted to calculate the library titer according to the formula: pfu/mL = number of plaques × dilution factor × 10^3^ μL/mL (μL of diluted phage plated). The recombination efficiency was identified by blue/white screening in *E.coli* XL1-Blue. Colony PCR was used to confirm the size of inserted fragments in the library. After amplification, the completed cDNA libraries were stored in 7% dimethyl sulfoxide at −80 °C.

### 4.5. Sequence Analysis Method

cDNA clones were selected randomly from the cDNA library and single-pass sequenced at the 5′ end on an ABI 3730 Genetic Analyzer (Applied Biosystems, Foster City, CA, USA). A large-scale EST sequencing project for Bengal tiger was initiated to identify and functionally annotate as many unique transcripts as possible. The processed cDNA sequences were used to perform the BLAST search at the GenBank database to compare all available ESTs and genes to date [21]. BLASTX results with bit scores greater than 80 and *E*-values of less than 10^−10^ were generally regarded as significant match [22–24]. According to sequencing results of the Bengal tiger cDNA library, 4 ferritin EST sequences were obtained and assembled the full-length ferritin cDNA. The ferritin coding regions and domains were predicted online with ORFfinder and SMART [22].

### 4.6. Expression and Purification of pET32a-TAT-Ferritin

TAT protein transduction domain was designed and fused to the N-terminus of Ferritin by PCR method, using specific primers (Ferritin sense: CCA TGG CTT ATG GTC GTA AAA AAC GTC GTC AGC GTC GTC GTA TGA CGA CCG CAT CCC CCT CG and Ferritin antisense: CTCGAG ACT GTC ACT GTG TTT CAG GGT). The amplified fragment was inserted into pET32a expression vector (Novagen, Darmstadt, Germany) N*co* I–X*ho* I sites and transferred into BL21 (DE3) cells. The recombinant protein TAT-Ferritin was expressed in BL21 cells by induction with IPTG at 1–8 h. The expression condition was optimized, soluble proteins were obtained using BugBuster protein extraction reagent (Novagen), purification of proteins were performed by Ni–NTA His•Bind affinity chromatography, the final protein was sterilized by filtration with 0.2 μm of filter membrane and quantitated using BCA assay.

## 5. Conclusions

In conclusion, by cryopreserving nationally protected Bengal tiger cells, we have generated an important genomic resource that captures the genomic information of this endangered breed at the cell level. Second, the first high-quality full-length cDNA library of Bengal tiger had been constructed and offered an efficient way to identify more genes of this majestic species. Meanwhile, expression and purification of Trx-TAT-Ferritin fusion protein showed quality and stability of the cDNA library.
